# Mechanical Recovery of Damaged Glass Fiber Reinforced Polypropylene via Repetitive Sub-Melting Thermal Annealing: A Molecular and Structural Study

**DOI:** 10.3390/polym18192441

**Published:** 2026-10-07

**Authors:** Tetsuo Takayama, Jun Funabashi

**Affiliations:** 1 Graduate School of Organic Materials Science, Yamagata University, Yamagata 992-8510, Japan; 2 Department of Polymeric and Organic Materials Engineering, Yamagata University, Yamagata 992-8510, Japan

**Keywords:** crystallinity, glass fiber reinforced thermoplastics (GFRTP), interfacial debonding, polypropylene, reptation theory, thermal repair

## Abstract

This study clarifies the mechanical recovery and structural retention of damaged glass fiber-reinforced polypropylene (PP/GF) composites subjected to repetitive sub-melting thermal annealing at 160 °C. Initial thermal annealing increased matrix crystallinity from 0.30 to 0.37 (+23.3%), thereby elevating flexural strength from 103 MPa to 122 MPa (+18.4%). Following one repair cycle, flexural strength was completely restored to 103–105 MPa (100% recovery rate). However, after three repair cycles, short-duration annealing (120 s) resulted in severe mechanical degradation down to 30 MPa (−70.9%), whereas long-duration annealing (1800 s) retained a strength of 59 MPa (−42.7%, preserving 57.3% of initial strength). Concurrently, the crystallinity index increased to 0.49 (+63.3%) for 1800 s. These results establish that extended dwell time at sub-melting temperatures is essential for promoting molecular chain re-entanglement while suppressing void growth. These findings present an engineering guideline for low-energy, structure-preserving repair of thermoplastic composites.

## 1. Introduction

In modern industrial sectors, weight reduction in structural bodies in automotive, aerospace, and transport applications is an urgent priority to mitigate environmental impact and reduce carbon dioxide emissions. To meet these demands, glass fiber-reinforced thermoplastics (GFRTP) and carbon fiber-reinforced thermoplastics (CFRTP) are attracting significant attention as lightweight alternatives to traditional thermosets [1,2]. As emphasized by Friedrich and Almajid, optimizing thermoplastic composite manufacturing plays a decisive role in mass-production sectors by shortening cycle times and lowering costs [1]. Furthermore, Thomason demonstrated that the mechanical properties of injection-molded PP/GF are highly sensitive to internal fiber length (aspect ratio) and fiber concentration, making structure-preserving design an indispensable necessity for practical implementation [2]. 

Unlike thermosetting polymers that form permanent three-dimensional covalent networks, thermoplastics consist of non-covalently bonded molecular chains that reversibly soften, flow, and solidify upon heating above their glass transition (T_g_) or melting temperature (T_m_) [3]. Traditional repairs for thermoset composites rely on adhesive bonding with patch materials or mechanical joining (bolts/rivets), which inherently introduce stress concentrations, weight penalties, and galvanic corrosion risks [4,5]. Conversely, physical mechanical recycling of GFRTP via re-melting generates strong mechanical shearing forces that break glass fibers, leading to severe fatigue strength degradation and forcing materials into low-value “cascade recycling” [6,7]. Therefore, non-destructive repair methods that bypass re-melting and directly restore accumulated micro-damage while maintaining component geometry are strongly required.

Therefore, a novel approach is strongly required: that approach must bypass the fiber-breakage-inducing re-melting process, maintain both component shape and fiber length, and actively restore degraded mechanical properties. This study specifically examines the microscopic structural damage accumulated within the material before it reaches ultimate failure.

The microscopic structural damage accumulated prior to ultimate failure includes localized matrix plastic deformation, matrix crazing theorized by Kramer and Berger [8], and fiber–matrix interfacial debonding formulated by Clyne and Hull [9]. For millimeter-scale macrocracks, advanced integrated mechanisms such as “Close-Then-Heal” utilize shape memory spring-back forces to fuse crack interfaces [10]. Additionally, chemical self-healing polymer systems—such as microencapsulation or autonomous supramolecular networks—exhibit excellent healing efficiency, but face daunting barriers in load-bearing structural composites due to high monomer costs, expensive catalysts, and reduced thermal/mechanical stiffness [11,12].

In contrast, physical thermal annealing requires no chemical modification or repair additives, relying solely on thermal energy input to drive physical relaxation, interfacial contact, and chain re-entanglement. This physical healing dynamic is described by De Gennes’ reptation theory [13], wherein polymer chains escape their spatially constrained tubes via Brownian motion to regenerate entanglement density across damaged interfaces, as formulated by Wool and O’Connor’s crack healing model (σ(t)/σ_∞_∝t^1/4^) [14]. Heating near T_m_ activates micro-Brownian motion of amorphous chains, promoting residual stress relaxation, craze closure via free-volume redistribution, and secondary crystallization.

However, a major engineering bottleneck in thermal repair is “deconsolidation”—uncontrolled bulk deformation and internal void expansion under thermal loading [15,16]. To suppress deconsolidation, local thermal stimulation techniques such as non-contact magnetic induction heating and nanocomposite resistance welding have been investigated, often integrated with structural health monitoring (SHM) for smart maintenance [17]. Furthermore, recent advances in thermoplastic composite restoration emphasize the necessity of non-destructive, energy-efficient thermal processing [18,19].

Despite these advancements, the fundamental interplay between molecular reptation dynamics, crystalline skeleton retention, and structural anisotropy during repetitive healing cycles remains insufficiently understood. Specifically, how reptation under crystalline constraints governs the trade-off between reversible matrix recovery and irreversible interfacial debonding across multi-cycle maintenance has not been systematically elucidated. Addressing this gap requires an integrated molecular and structural study that correlates macro-scale mechanical performance with micro-scale matrix orientation and fiber alignment.

To address these issues, this study systematically investigates the mechanical recovery and structural evolution of damaged PP/GF composites subjected to up to three repetitive sub-melting thermal annealing cycles at 160 °C (T_m_ ≒ 170 °C) under short (120 s) and extended (1800 s) durations. Through three-point bending tests (ISO 178:2019 [20]), 3D fiber orientation mapping via micro-focus X-ray CT, and matrix crystallinity/molecular orientation via ATR FT-IR, we establish the molecular mechanisms and practical engineering boundaries for low-energy, structure-preserving repair of commodity thermoplastic composites.

## 2. Materials and Methods

### 2.1. Materials and Pellet Preparation

Commercially available glass fiber-reinforced polypropylene pellets (PP/GF; PG6N1; Daicel Polymer Ltd., Tokyo, Japan) with a nominal fiber content of 30 wt% were used as received. Because the materials were acquired as fully compounded commercial pellets, specific proprietary formulation details—such as the exact melt flow index (MFI), stereoregularity (tacticity), polydispersity, and precise silane coupling agent chemistry—were not disclosed by the manufacturer. For theoretical estimation of molecular mobility and reptation behavior, a representative weight-average molecular weight (M_w_) of 3.0 × 10^5^ g/mol and a typical fiber diameter of 10–13 µm, which are standard for commercial injection-molding grade GF/PP composites, were adopted as baseline parameters [21].

As indicated by Thomason’s detailed investigations of injection-molded short and long fiber-reinforced polypropylene, mechanical properties of FRP, such as tensile strength and stiffness, are extremely sensitive to internal fiber length (aspect ratio) and fiber volume fraction [2]. The glass fibers used in this material are pre-treated with a silane coupling agent to ensure excellent interfacial affinity with the resin, forming a robust fiber–matrix interface in the initial as-molded state.

### 2.2. Specimen Molding Conditions

**Figure 1:**
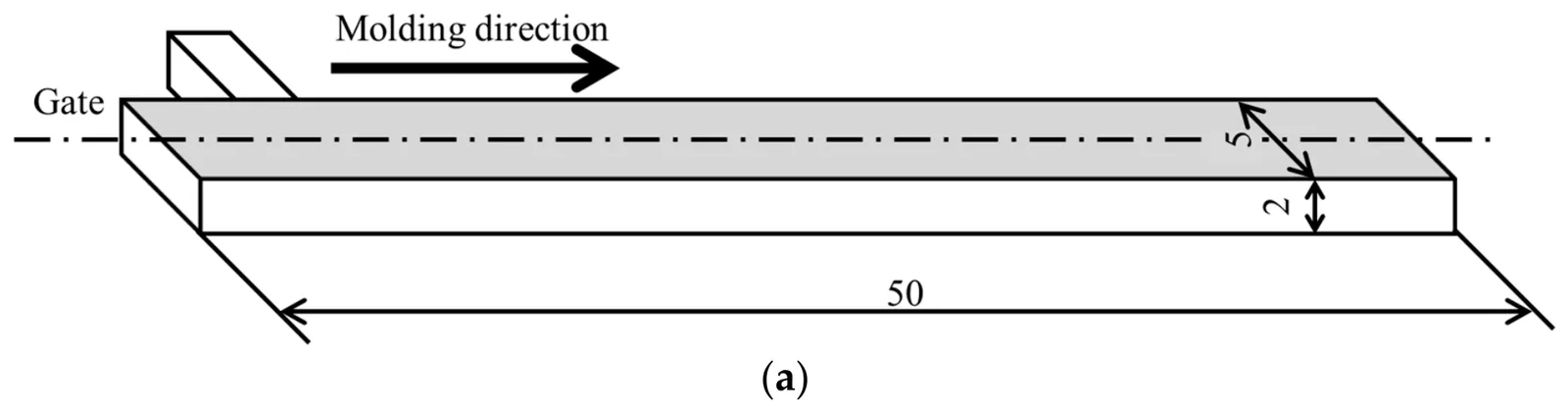

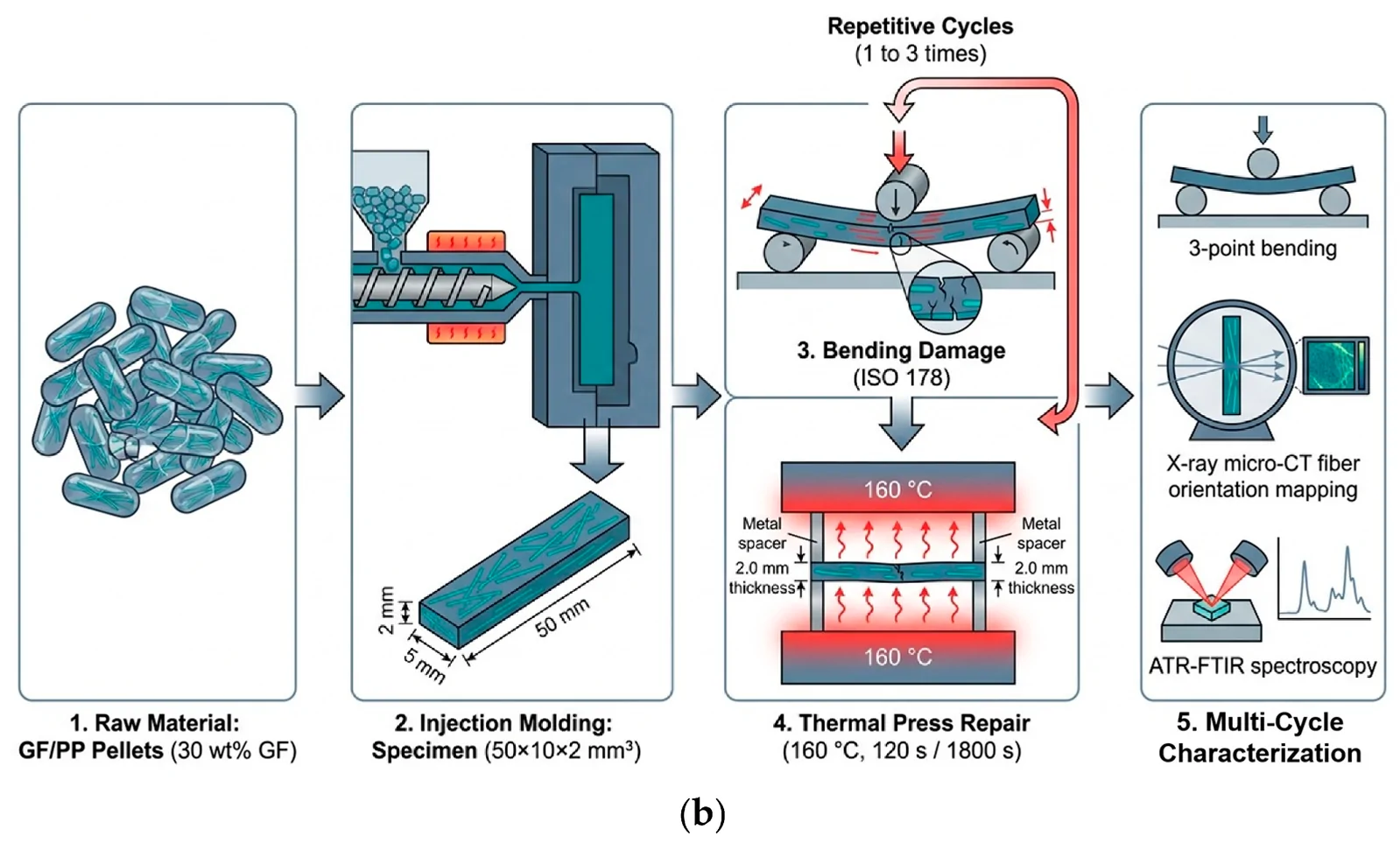
(**a**) Specimen dimensions and geometry for three-point bending tests (Unit: mm). (**b**) Schematic representation of the experimental workflow.

Using the PP/GF pellets, beam specimens (50 mm length, 10 mm width, 2 mm thickness) were molded using an ultra-compact electric injection molding machine (C, Mobile 0813; Shinko Sellbic Co., Ltd., Tokyo, Japan). The plunger diameter of the molding machine is 10 mm. Figure 1a portrays the shape of the injection-molded part, and Figure 1b portrays schematic representation of the experimental workflow, including raw GF/PP pellet materials, injection-molding of test specimens, displacement-controlled damage introduction (ISO 178 [20]), repetitive sub-melting thermal press repair at 160 °C under controlled metal spacing, and multi-cycle structural and mechanical characterizations. Specific molding parameters were set to a cylinder temperature of 200 °C, mold temperature of 50 °C, and injection speed of 20 mm/s. To minimize surface sink marks and internal microvoids in the molded parts, the holding pressure after cavity filling was set to 70 MPa with a holding time of 45 s. After molding, the specimens were conditioned for 24 h under standard room conditions (23 °C, 50% relative humidity) before being subjected to various measurements and mechanical tests.

### 2.3. Damage Induction and Thermal Repair Process

To evaluate the mechanical properties and to introduce mechanical damage to the molded short-bar specimens, three-point bending tests were conducted in accordance with ISO 178:2019 [20]. This mechanical test was adopted because the load must be applied reliably to the exact same location during repeated cycles. Measurements were performed using a small universal mechanical testing system (MCT-2150; A&D Co., Ltd., Tokyo, Japan). Specimen dimensions were 50 mm (length) × 10 mm (width) × 2 mm (thickness) with a support span length of 40 mm and a crosshead speed of 10 mm/min. To avoid complete material fracture (ultimate failure) and loss of continuity while ensuring reproducible and highly irreversible internal damage (microcrazes and interfacial debonding), a displacement control scheme was established to stop loading forcibly when a pre-set maximum deflection reached a specific threshold.

From the measured load–deflection relation, the flexural stress–flexural strain curve was derived. Flexural modulus (E_f_) was calculated from the initial elastic slope. Also, the maximum flexural strength (σ_f_) was inferred from the peak stress. The flexural yield stress (σ_fy_) was defined as the stress corresponding to the maximum load or local peak on the stress–strain curve. To characterize the nonlinear lateral deformation behavior near the tension surface (skin layer) under flexural loading, the effective Poisson’s ratio at flexural yielding (υ_eff_) was determined. Rather than measuring microscopic elastic strain ratios (transverse to longitudinal strain) at infinitesimal deformations, this study evaluates the structural bulk response of the short-bar specimen at the onset of yielding. Based on the mechanical constitutive formulation for composite short beams under three-point bending proposed by Takayama and Oneda, υ_eff_ is calculated using Equation (1) [22].

(1) $$\upsilon_{\mathrm{eff}}=\frac{\sigma_{\mathrm{fy}}}{3E_{f}\varepsilon_{\mathrm{fy}}-\sigma_{\mathrm{fy}}}$$

In that equation, σ_fy_ denotes the flexural yield stress, E_f_ stands for the flexural modulus, and ε_fy_ represents the flexural yield strain. Poisson’s ratio obtained using this method represents the skin-layer vicinity Poisson’s ratio of the short-bar specimen. It must be emphasized that υ_eff_ derived from this yield-point formulation reflects the apparent, skin-layer vicinity Poisson’s ratio in the nonlinear elastoplastic regime, capturing both the viscoelastic response of the polymer matrix and the structural constraint imposed by the embedded fibers before macroscopic failure.

**Figure 2:**
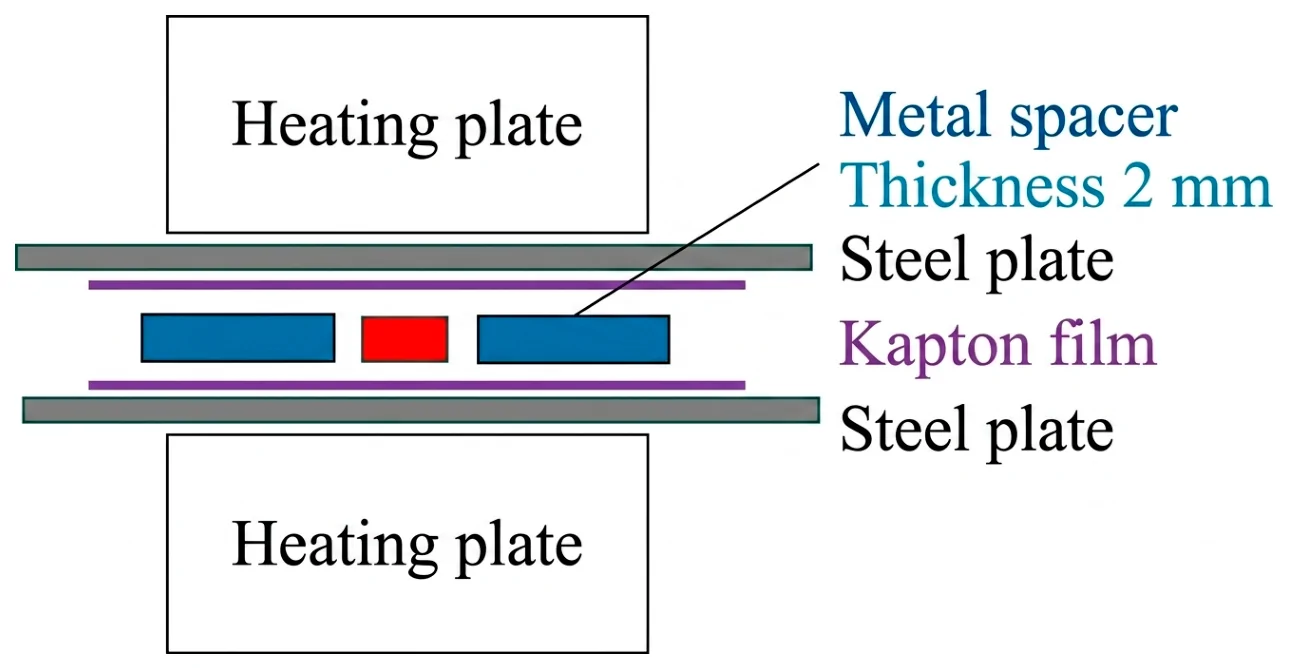
Schematic illustration of the hot-pressing mold assembly for sample preparation. Red color part means the sample.

As demonstrated by fracture mechanics studies of polymer crack healing and welding by Jud, Kausch, and Williams, mild external pressure during annealing plays a crucially important role in forcibly bringing thermal-flowing amorphous chains into contact with opposing crack surfaces or debonded fiber–matrix interfaces [23]. This pressure and contact promote physical rewetting, forming an essential physical precursor step for the reptation process. Thermal repair was performed using a vise thermal press apparatus (MNP-002D; AS One Corp., Osaka, Japan). The upper and lower heating plates were preheated to 160 °C. The actual specimen surface temperature was continuously monitored using a K-type thermocouple embedded in the heating plate, ensuring temperature stability within ±0.5 °C and minimal cross-sectional thermal gradient. Figure 2 portrays the hot-pressing mold assembly configuration. Metal spacers matching the specimen thickness (2.0 mm) were utilized to prevent geometric distortion. This heat press is equipped with a screw-type clamping mechanism, which makes it impossible to measure the pressure accurately. However, since a 2.0 mm metal spacer—equivalent to the thickness of the molded part—is inserted in this study, we have determined that the pressure exerted on the molded part is negligible. To prevent polymer adhesion to the press surface, the specimen was sandwiched between highly heat-resistant Kapton films from top and bottom, stacked between smooth iron plates, and subjected to uniaxial compressive pressure. The sample temperature was verified using an embedded thermocouple to ensure minimal thermal gradient across the thickness. After holding for 120 s or 1800 s, the test specimens were carefully removed from the heat press without disturbing the assembly and cooled to room temperature by air cooling. The repair durations of 120 s and 1800 s were selected based on preliminary kinetic calculations of PP chain relaxation times.

A sequence from “uniaxial bending damage induction” to “thermal press annealing repair” was defined as one cycle. Up to three repeated cycles were performed on the same specimen. To ensure measurement reproducibility and statistical reliability, testing and repair processes for each condition were conducted on at least three specimens. Mean values along with standard deviations were calculated for flexural strength, flexural modulus, and the effective Poisson’s ratio.

### 2.4. Fiber Orientation Measurement

To track the effects of the injection molding process and subsequent repetitive “damage–thermal repair” cycles on the 3D fiber orientation state inside the specimen, non-destructive structural observation was performed using a micro-focus X-ray CT scanner (ScanXmate-D225RSS270; Comscantecno Co., Ltd., Tokyo, Japan). Imaging was conducted with 70 kV tube voltage, 150 μA tube current, and 15× magnification to acquire high-resolution projection images of the target region.

Micro-CT analysis focused on the 100 µm thick outer skin layer in the central load-bearing region, where maximum bending stresses occur. Over 150 independent fibers were sampled randomly; preliminary sensitivity analysis confirmed that sampling over 150 fibers yields a statistically converged mean orientation angle. It should be noted that the spatial resolution limit of the present micro-CT scanner (approximately 2–3 µm) is insufficient to resolve sub-micron interfacial debonding or microvoids directly.

From transmitted slice images extracted along the plane formed by the molding direction (MD) and width direction (WD), individual glass fibers were segmented using image analysis software (WinROOF2023; Mitani Corp., Tokyo, Japan). Following the 3D fiber structure observation and image extraction protocol for short-fiber polymer composites based on micro-CT imaging established by Shen, Nutt, and Hull, over 150 independent glass fibers were sampled randomly from the central load-bearing region where the stress concentration is highest. The orientation angle (θ) of each fiber relative to the molding direction (MD) was measured to evaluate the mean fiber orientation angle and its orientation angle distribution (standard deviation) in the skin layer [24].

### 2.5. Fourier-Transform Infrared Spectroscopy (FT-IR)

To evaluate the microscopic effects of thermal annealing repair and its repetition on the hierarchical structure of the PP matrix (crystallinity and molecular chain orientation), spectroscopic analysis of the matrix polymer was performed using a Fourier-transform infrared spectrometer equipped with a single-reflection diamond attenuated total reflection crystal (FT-IR, Nicolet iS5; Thermo Fisher Scientific K.K., Waltham, MA, USA). Spectra were acquired in the wavenumber range of 650–4000 cm^−1^ with a spectral resolution of 4 cm^−1^ and 32 co-added scans. An automatic polynomial baseline correction was applied to all spectra. The absorbance peak heights at 998 cm^−1^ (crystalline polypropylene helical phase) and 973 cm^−1^ (reference band) were utilized to determine the relative crystallinity index and dichroic ratio. Polarized infrared light was irradiated parallel to the molding direction (MD) and width direction (WD) to obtain the respective polarized spectra.

In accordance with the classical approach for deformation-induced crystallization behavior and orientation anisotropy analysis using polarized ATR-FTIR spectroscopy by Siesler et al., the dichroic ratio (R), and Hermans orientation index (S) (which indicates the degree of polymer chain orientation) were calculated based on the absorbance intensity at specific vibrational wavenumbers from the polarized absorption spectra [25]. The Hermans orientation index S is calculated using the following Equation (2).

(2) $$S=\frac{R-1}{R+2}$$

In that equation, R represents the dichroic ratio defined by Equation (3), representing the ratio of polarized absorbances in the molding direction (MD) to the width direction (WD) at a specific absorption band.

(3) $$R=\frac{A_{\mathrm{MD}}}{A_{\mathrm{WD}}}$$

Therein, A_MD_ and A_WD_ denote infrared absorbances at wavenumbers sensitive to uniaxial orientation of molecular chains. For this evaluation, the crystalline absorption peak at 998 cm^−1^, corresponding to the crystalline-sensitive band, was adopted.

Furthermore, based on the quantitative phase structure evaluation method for polymer crystalline and amorphous regions via infrared spectroscopy theorized by Fanconi et al., the crystallinity index (X_c_), describing phase structure changes in the PP matrix, was determined using Equation (4) based on the absorbances of crystalline-sensitive and crystalline-insensitive bands [26].

(4) $$X_{c}=\frac{A_{998}}{A_{973}+A_{998}}$$

In this evaluation, the ratio of the crystalline absorption peak at 998 cm^−1^ (crystalline-sensitive band originating from the regular arrangement/helical crystalline lamellae of polypropylene) to the reference peak at 973 cm^−1^ (amorphous/reference band originating from backbone chain stretching, largely unaffected by crystallization state) was adopted. It is noteworthy that X_c_ calculated via Equation (4) represents a relative crystallinity index based on infrared absorbance peak ratios. Although this spectroscopic index directly reflects structural changes in helical crystalline sequences of the PP backbone, it differs in magnitude from the absolute mass/volume degree of crystallinity derived from DSC enthalpy measurements (ΔH_m_/ΔH_m0_). For this study, X_c_ is used as a relative structural parameter to track the progress of lamellar crystallization across thermal repair cycles. To ensure high reproducibility, ATR spectral measurements were repeated five times at different points on each sample. The mean values and standard deviations of the Hermans orientation index and crystallinity index were calculated statistically.

### 2.6. Thermal Property Evaluation

To clarify the thermal properties of the PP/GF composite, particularly the thermal stability of the crystalline phase and the melting temperature range (repair temperature window), heat flux evaluation was conducted using a differential scanning calorimeter (DSC, Q-2000; TA Instruments, New Castle, DE, USA). Measurements were executed in accordance with general thermal analysis principles specified in ISO 11357-1:2023 [27]. To obtain representative bulk values free from localized hierarchical structural variations, a micro-sample (mass: approximately 5 mg) was sectioned from the molded PP/GF specimen and was sealed in an aluminum pan. Under a nitrogen atmosphere (flow rate 50 mL/min), a first heating scan (1st Heating) was performed from 40 °C to 220 °C at a heating rate of 10 °C/min, and the resulting DSC thermogram was recorded. First-heating scans were specifically evaluated because the core objective of this study is to analyze the thermal repair behavior under the actual processing- and damage-induced thermal history (crystalline skeleton) of the as-molded composite. For comparison, second-heating scans were also performed following controlled cooling at 10 °C/min to confirm the intrinsic melting behavior of the PP matrix independent of thermo-mechanical history. Based on the polymer thermodynamics of polypropylene crystal growth modes, oriented crystallization, and crystalline lamella melting behavior by Monasse and Haudin, evaluating these physical melting onset behaviors established the theoretical validity for setting the annealing temperature (160 °C). This condition activates micro-Brownian motion and thermal entanglement reconstruction of amorphous chains selectively while highly preserving the injection-molded bulk geometry (crystalline framework) [28].

## 3. Results

### 3.1. Thermal Properties of PP/GF

**Figure 3:**
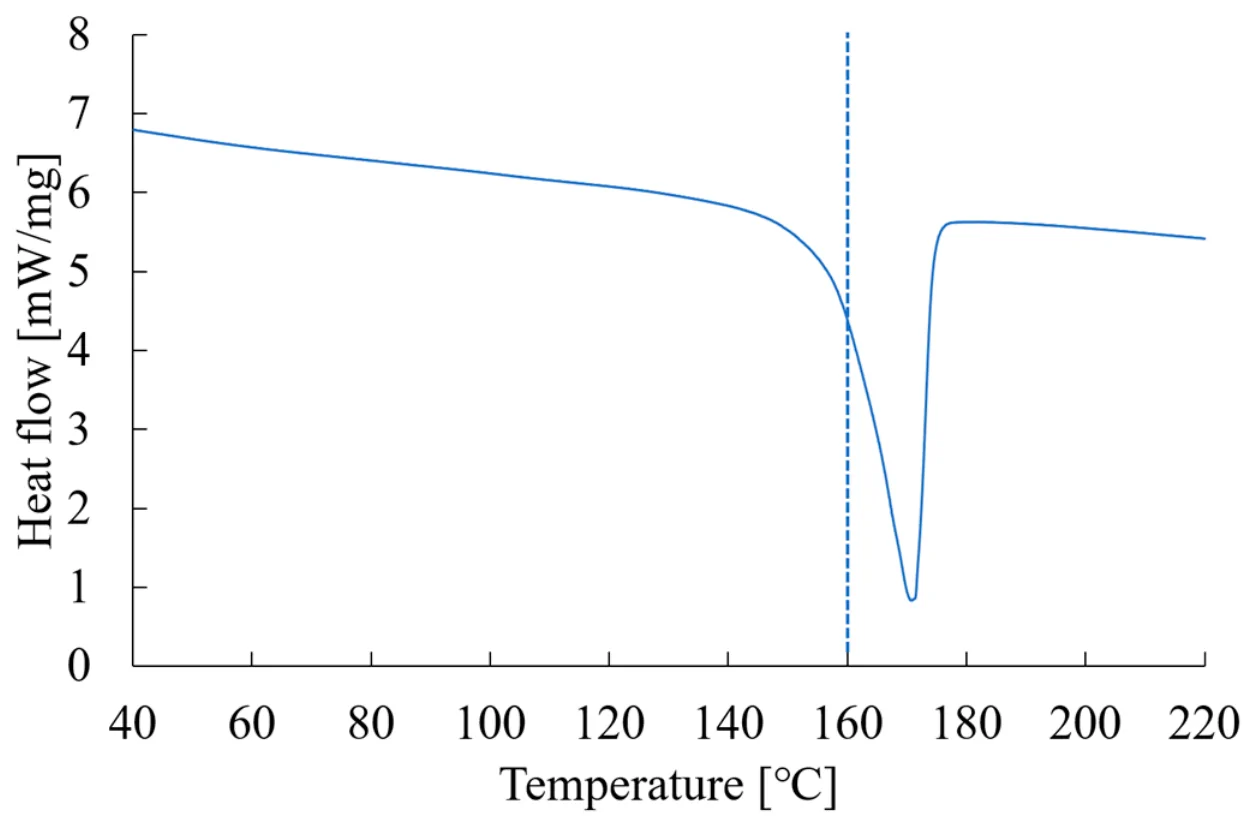
First-heating DSC thermogram of the as-molded PP/GF composite matrix at a heating rate of 10 °C/min. The vertical dashed line shows the target thermal repair temperature (160 °C).

Differential scanning calorimetry (DSC) was conducted to investigate the thermal properties of the PP/GF composite material and their correlation with thermal healing behavior. The DSC thermogram is depicted in Figure 3. The vertical dashed line shows the target thermal repair temperature (160 °C).

In the DSC thermogram, a gentle endothermic rise began at approximately 160 °C for the PP/GF composite material with a subsequent a clear, sharp endothermic peak top corresponding to crystal melting observed at approximately 170 °C. This melting behavior clearly reflects the thermal transition of the crystalline lamellar structure of polypropylene (PP): the semi-crystalline polymer used as the matrix. According to thermodynamic insights into the melting and recrystallization behavior of polypropylene crystals reported by Monasse and Haudin, the melting point (170 °C) represents the temperature at which most of the crystal lamellae melt and undergo a phase transition to a thermodynamically random coil (isotropic melt state) [28]. In contrast, the selected repair temperature of 160 °C lies within the broad endothermic melting onset region (spanning approximately 145 °C to 175 °C). At 160 °C, only a minor fraction of thinner or defective crystalline lamellae undergoes localized partial melting (estimated at <5% of the total melting enthalpy), which expands the free volume of amorphous chains and activates local micro-Brownian segmental mobility. Concurrently, the primary robust crystalline framework remains un-melted, effectively providing structural support to prevent self-weight deformation (deconsolidation). Therefore, 160 °C represents an optimal sub-melting thermal window that balances localized interfacial molecular mobility with macroscopic geometric stability.

### 3.2. Initial Structural Analysis of Molded Specimens

#### 3.2.1. Fiber Orientation State (X-Ray CT)

**Figure 4:**
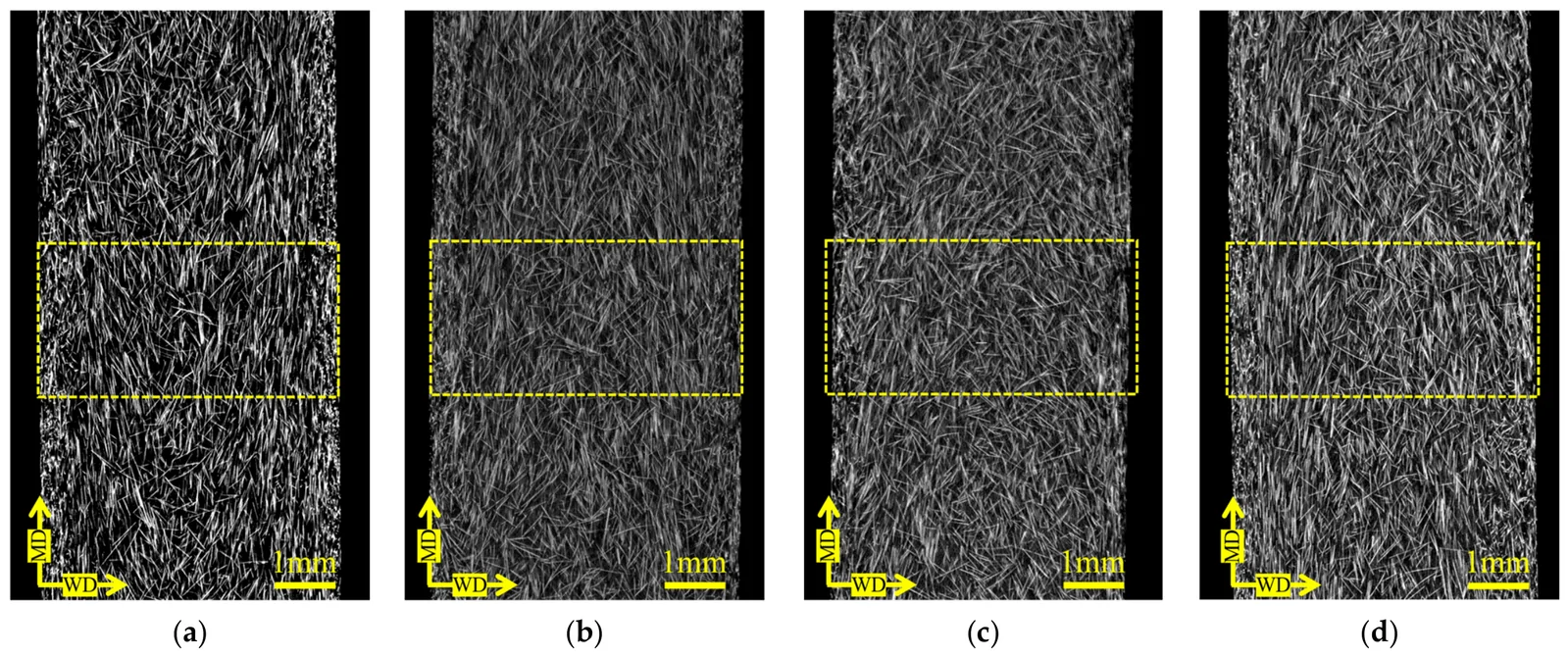
X-ray computed tomography (CT) images showing the internal glass fiber orientation and distribution in injection-molded PP/GF composites across sequential repair cycles: (**a**) as-molded state, (**b**) after the first repair cycle for 120 s, (**c**) after the second repair cycle for 120 s, and (**d**) after the third repair cycle for 120 s. Yellow arrows in the lower left of each panel indicate the molding direction (MD) and width direction (WD). Yellow dashed rectangles encompass the respective target localized repair zones.

To track the effects of cavity filling behavior during injection molding as well as the subsequent repetition of “damage–thermal repair” annealing on the three-dimensional geometric arrangement (orientation structure) of glass fibers within the skin layer of the specimen, non-destructive observation was conducted using microfocus X-ray CT. Figure 4 presents results obtained from observing the fiber orientation of the PP/GF injection-molded specimens using X-ray CT. This observation was performed only for the as-molded specimen and the specimen thermally repaired under the condition of 160 °C for 120 s. The area enclosed by the dotted line in the figure corresponds to the region subjected to load in the three-point bending test described later. In the skin layer region (the region from the surface to a depth of approximately 100 μm) of the untreated specimen (as-molded) shown in Figure 4a, an anisotropic structure highly uniaxial-oriented along the resin flow direction (MD: Molding Direction) was observed because of strong shear flow near the mold wall during injection molding.

In the region subjected to the three-point bending load (yellow dotted line), no noticeable change was observed in the overall orientation direction or distribution state of the glass fibers in any sample after one thermal repair (Figure 4b), two thermal repairs (Figure 4c), or 3 thermal repairs (Figure 4d). No prominent localized fiber rearrangement or non-uniform alignment disorder accompanied by an increase in thermal repair cycles was observed. A stable orientation structure relative to the flow direction (MD) was maintained at each repair stage.

**Figure 5:**
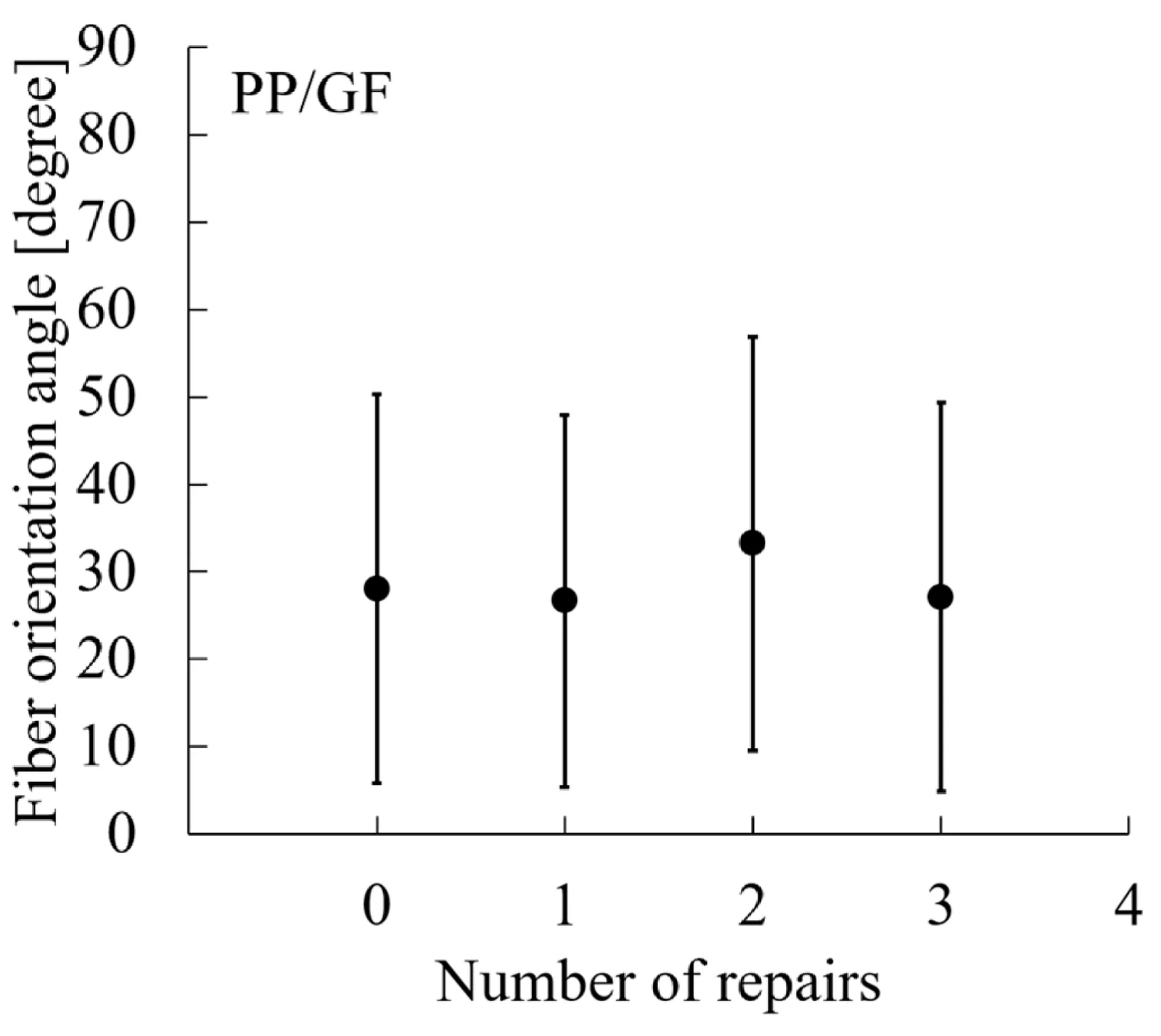
Glass fiber orientation angle in the PP/GF composite as a function of the number of thermal repair cycles. A value of “0” on the horizontal axis denotes the as-molded specimen before thermal repair.

Following the image analysis approach for geometric spatial orientation of short-fiber-reinforced composites by Fischer and Eyerer, as well as the short-fiber segmentation extraction protocol based on micro-CT imaging proposed by Shen, Nutt, and Hull, more than 150 independent fibers were selected randomly from the central load region, where stress is most concentrated, to measure the average orientation angle θ [22,29]. Figure 5 presents the evaluation results of the fiber orientation distribution in the skin layer region of the PP/GF injection-molded specimens.

The mean fiber orientation angles for cycles 0, 1, 2, and 3 were 28 ± 12°, 27 ± 12°, 33 ± 13°, and 27 ± 12°, respectively. One-way ANOVA confirmed no statistically significant difference among the repair cycles (*p* > 0.05), validating that macroscopic fiber alignment remained stable. At each repair stage, the average value remained within an extremely narrow range of 27–33°: no systematic increase/decrease pattern, disruption of alignment structure, or partial rearrangement behavior was observed.

Furthermore, regarding the orientation angle distribution range (error bars indicating standard deviation), a constant and stable distribution width of approximately 5° to 55° was maintained throughout all cycle repetitions. This highly precise orientation stability indicates that, because the thermal pressing process at 160 °C is strictly controlled in the partial melting region below the melting point of PP (170 °C), the primary crystalline lamellae constituting the higher-order structure constrain the morphological skeleton of the entire material, consequently preventing macroscopic thermal melt flow (deconsolidation) of the bulk resin.

As a result, because macroscopic flow stress (shear stress) is not generated under annealing, it was demonstrated that localized damage can be thermally closed selectively without disturbing the anisotropic fiber orientation structure controlled during initial injection molding at all.

#### 3.2.2. Hierarchical Structural Changes in PP Matrix via Thermal Repair (FT-IR)

To evaluate the effects of thermal annealing repair and its repetition on the hierarchical structure of the PP matrix, the matrix crystallinity index (X_c_) and Hermans orientation index (S) were calculated from ATR-FT-IR spectra. While differential scanning calorimetry (DSC) measures the volumetric bulk-average crystallinity integrated across the entire 2.0 mm specimen thickness, ATR-FTIR spectroscopy specifically probes the localized surface skin layer at an optical penetration depth of approximately 1–2 µm. In three-point bending deformation, maximum tensile/compressive stresses, localized plastic yielding, and matrix micro-crazing are heavily concentrated at the outermost skin layer. Consequently, ATR-FTIR serves as a highly surface-sensitive structural parameter that directly governs and correlates with the flexural strength recovery and craze healing behavior evaluated in this study.

**Figure 6:**
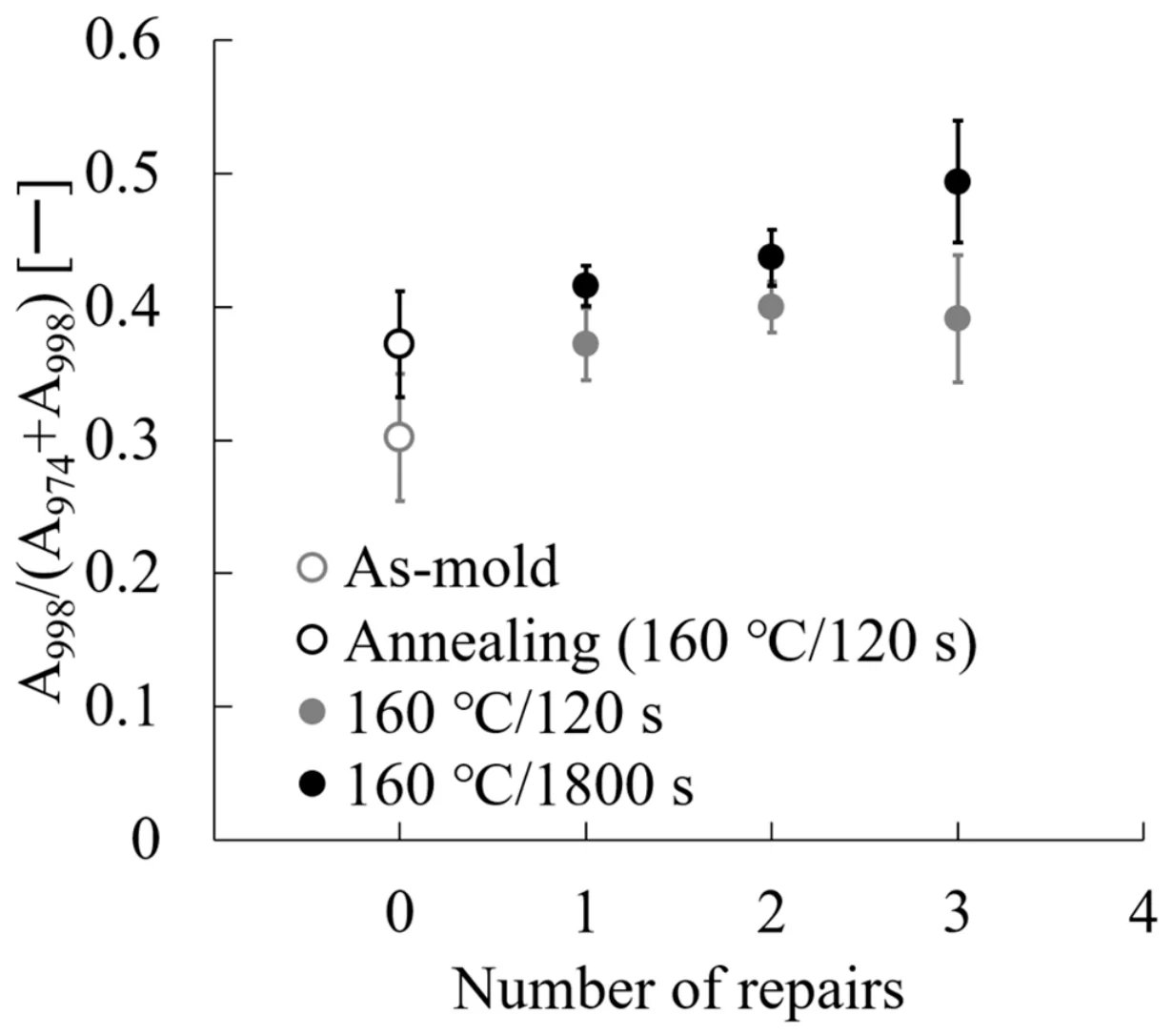
Relative crystallinity index A_998_/(A_974_ + A_998_) of the polypropylene matrix in the PP/GF composite evaluated by Fourier transform infrared (FTIR) spectroscopy as a function of the number of thermal repair cycles.

Figure 6 portrays the relation between the crystallinity index and the number of thermal repairs. The error bars in the figure represent the standard deviation. The matrix crystallinity index (X_c_) in the as-molded (untreated) state was 0.30. Upon applying thermal annealing at 160 °C for 120 s, X_c_ increased to 0.37. For specimens repaired under the 120 s condition, X_c_ reached 0.37 after the first repair and maintained nearly constant values of 0.40, 0.39, and 0.39, respectively, after the second and third repair cycles. By contrast, under the extended annealing condition of 1800 s, X_c_ exhibited a continuous and marked increase with the number of repair cycles: 0.42 after the first repair, 0.44 after the second repair, and 0.49 after the third repair. This increase indicates that extended annealing strongly promotes lamellar crystal growth and phase structure reorganization.

**Figure 7:**
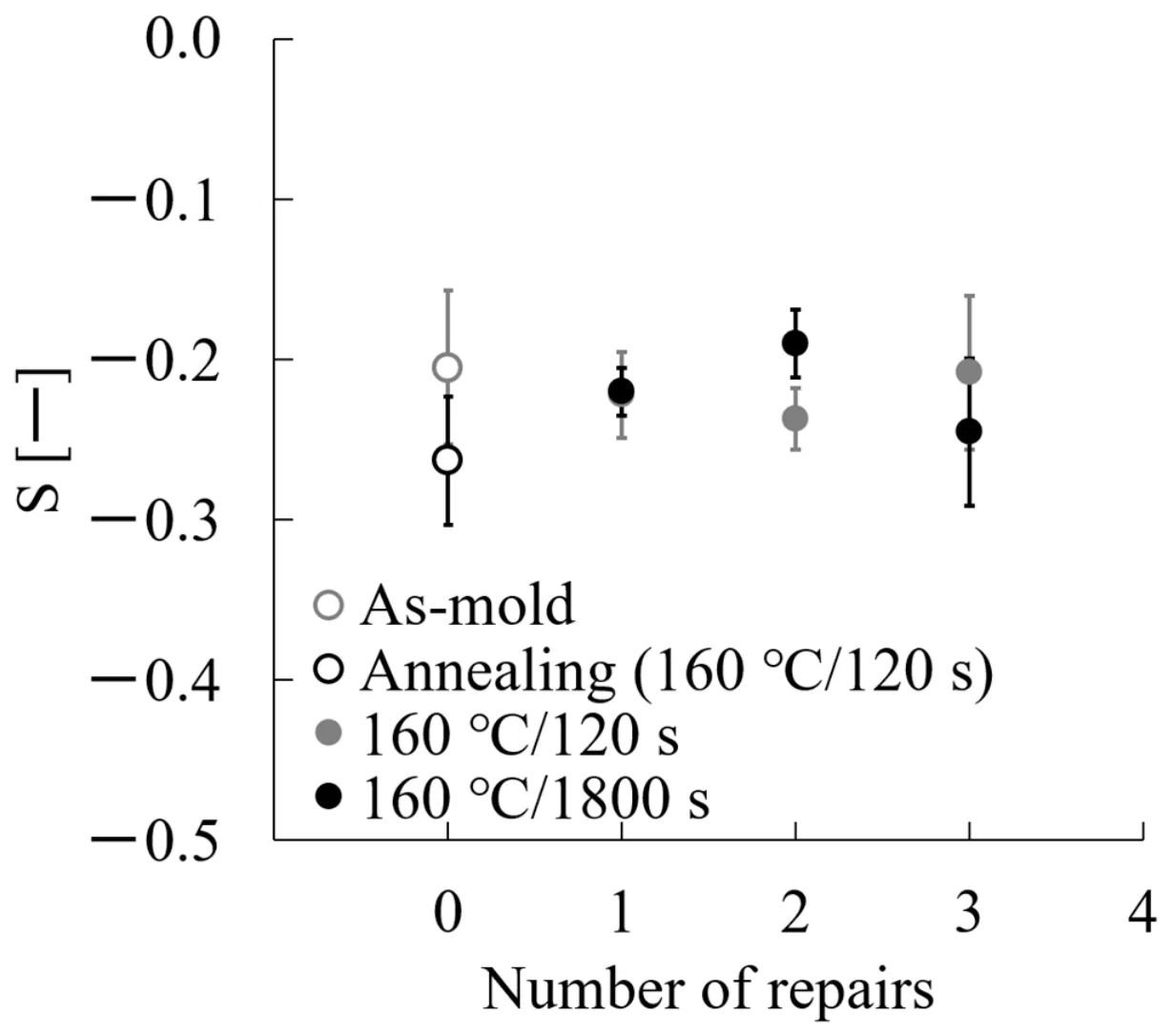
Hermans orientation factor (S) of the polypropylene (PP) matrix in the PP/GF composite as a function of the number of thermal repair cycles.

To assess changes in molecular chain alignment accompanied by thermal repair, the Hermans orientation index (S) was evaluated as a function of repair cycles. Figure 7 presents the relation between S and the number of thermal repairs. The error bars in the figure represent the standard deviation. The as-molded specimen exhibited an initial orientation index of S = −0.21, whereas the sample annealed at 160 °C for 120 s showed S = −0.26. For specimens repaired under the 120 s condition, S maintained nearly constant values of −0.22, −0.24, and −0.21, respectively, after the first, second, and third repair cycles. Similarly, under the 1800 s repair condition, S was −0.22 after the first cycle and −0.25 after the second and third cycles. Across all cycles and repair durations, no systematic trend or significant change in S was observed, with values remaining stable within the experimental uncertainty range (−0.19 to −0.26).

These results demonstrate that the molecular chain orientation (S) remains exceptionally stable within a narrow range (−0.19 to −0.26), revealing that macro-scale molecular alignment anisotropy is highly preserved without being erased by thermal relaxation. This preserved anisotropy proves that sub-melting localized thermal activation provides an effective processing window capable of enhancing matrix crystallinity while simultaneously preserving molecular orientation anisotropy: the origin of anisotropic mechanical performance in molded composites.

### 3.3. Mechanical Property Changes Associated with Repetitive “Damage–Thermal Repair” Cycles

**Figure 8:**
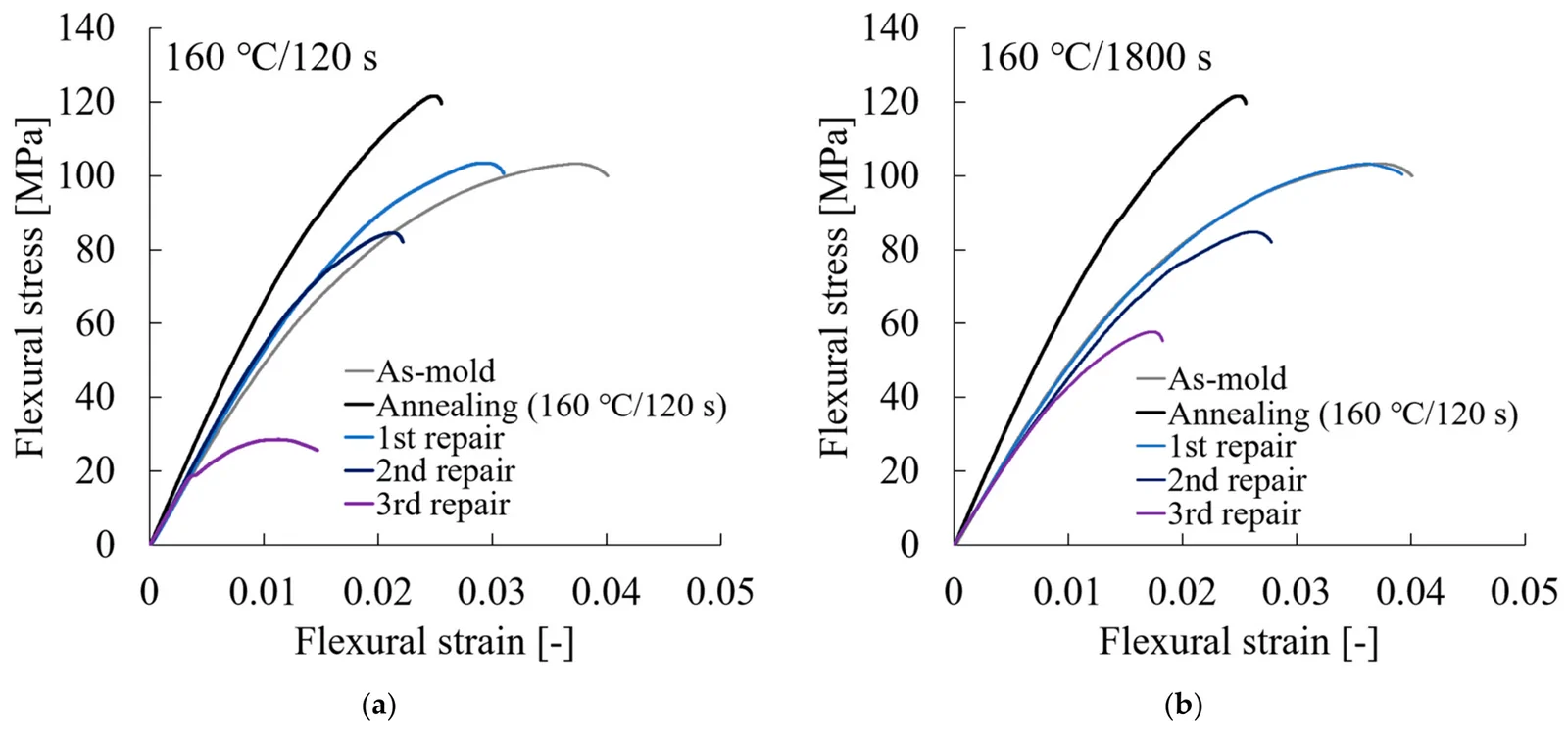
Flexural stress–strain curves of the PP/GF composite subjected to sequential thermal repair cycles under different heating durations: (**a**) repair at 160 °C for 120 s per cycle, and (**b**) repair at 160 °C for 1800 s per cycle.

**Figure 9:**
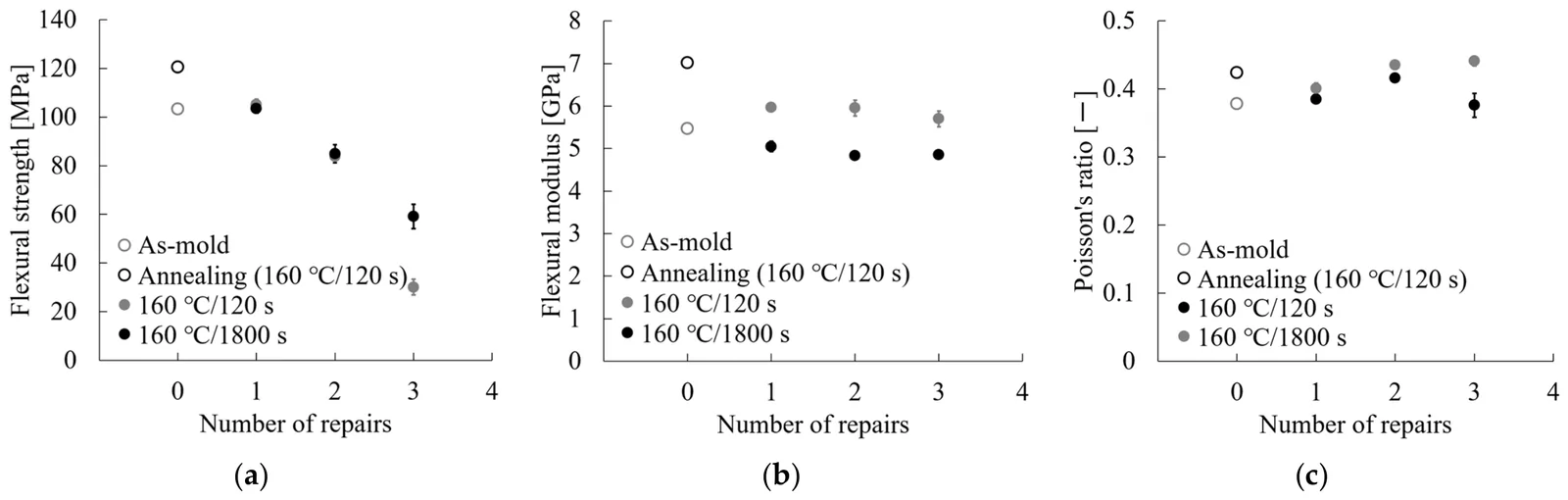
Mechanical properties of the PP/GF composite as a function of the number of thermal repair cycles under different heating durations: (**a**) flexural strength, (**b**) flexural modulus, and (**c**) Poisson’s ratio.

To evaluate the effects of thermal repair and annealing treatments on the mechanical properties of the material, flexural stress–flexural strain behavior was measured under different repair duration conditions (120 s and 1800 s). Figure 8 portrays examples of flexural stress–flexural strain curves. Figure 9 presents the behaviors of the Poisson’s ratio, flexural modulus, and flexural strength against the number of repair cycles obtained from the three-point bending tests.

The untreated material (as-molded) exhibited maximum flexural stress (flexural strength) of approximately 103 MPa, a flexural modulus of 5.5 GPa, and strain at breaking of approximately 0.040. By contrast, regarding the specimen subjected to annealing (flexural pretreatment) at 160 °C for 120 s (annealed), the flexural modulus increased sharply to 7.0 GPa because of the stiffness-enhancing effect accompanying thermal crystallization (increase in crystallinity index). Correspondingly, the flexural strength increased markedly to approximately 122 MPa. However, because of the suppression of amorphous chain mobility resulting from physical constraint by matrix crystalline lamellae (crystalline constraint effect), the strain at break decreased drastically to approximately 0.025, exhibiting a typical cold-crystallization behavior by which material properties shift toward high rigidity and low brittleness.

In the thermal repair cycles interspersed with large-deformation displacement-controlled damage via three-point bending, both maximum flexural stress and strain at breaking exhibited a tendency to decrease stepwise with an increasing number of repair cycles.

After the first thermal repair (1st repair), the maximum flexural stress-maintained values of 103–105 MPa under both 120 s and 1800 s conditions, fully recovering mechanical strength to a level equivalent to the initial properties of the untreated material (as-molded). This result confirms that, under partial-melting thermal stimulation at 160 °C, interdiffusion and re-entanglement of polymer chains at damaged interfaces proceeded sufficiently at the first repair stage for both duration conditions.

However, repeating the cycles led to dramatic divergence in mechanical response because of differences in the repair duration. Under the short-duration condition (120 s), flexural strength decreased to approximately 84 MPa and strain at breaking to approximately 0.030 after the second repair. After the third repair, the maximum flexural stress severely degraded to approximately 28–30 MPa. This degradation in mechanical properties is regarded as attributable to the following, while the crystallinity index reached a plateau near 0.39 early on, the heating retention time was decisively insufficient relative to the effective reptation time extended under crystalline constraint. Consequently, deep interdiffusion of polymer chains and re-adhesive flow at debonded glass fiber–matrix interfaces ended at an incomplete initial stage, leading unrepaired defects (voids) to accumulate within the material with each cycle.

By contrast, under the long-duration condition (1800 s), strength of approximately 85 MPa was maintained after the second repair. Even after the third repair, high mechanical properties were retained successfully, with maximum flexural stress of approximately 58–59 MPa and a flexural modulus of 4.8–5.1 GPa. This is true, probably because securing a sufficient thermal treatment duration of 1800 s (30 min) allowed the reptation motion of amorphous chains to proceed deeply, even in an environment in which mobility was delayed because of an increase in degree of crystallinity up to 0.49. Consequently, the proceeding reptation motion promotes rewetting and physical re-adhesion of flowing PP into cracks and debonded fiber interfaces to the deeper region and suppresses the accumulation rate of structural defects to an extreme limit.

Regarding the Poisson’s ratio, the as-molded sample showed 0.38 and the Annealed sample showed 0.42, but the thermal repair samples maintained almost constant values at 0.38–0.44, irrespective of the number of repairs or thermal treatment duration. From a mechanical physics perspective, this fact proves strongly that even when the sub-melting point thermal repair process proposed in this study is applied multiple times repeatedly, microscopic strength defects can be repaired selectively and repeatedly without compromising the inherent macroscopic elastic deformation behavior or lateral elastic deformation (volumetric expansion characteristics) of the composite material.

## 4. Discussion

### 4.1. Progression of Crystallization via Thermal Annealing and Improvement of Initial Mechanical Properties

The FT-IR measurements conducted for this study revealed the crystallinity index of the untreated specimen (as-molded) as 0.30, whereas that of the specimen subjected to annealing treatment (mere heat treatment) at 160 °C for 120 s increased markedly to 0.37. Concurrently, the maximum flexural stress rose from the initial value of approximately 103 MPa to approximately 122 MPa. The flexural modulus also showed a remarkable increase from 5.5 GPa to 7.0 GPa. This improvement in initial stiffness and strength is attributed to the annealing treatment at 160 °C, just below the melting point, locally activating the mobility of the PP polymer chains as the crystalline matrix and rapidly inducing recrystallization (cold crystallization and thickening of crystal lamellae). This phenomenon shows good agreement with insights into the heat treatment behavior of composite materials reported by Hull et al. [30].

From the perspective of higher-order structure changes, an increase in the degree of crystallinity raises the volume fraction of the rigid crystalline phase (crystalline lamellae) inside the material, markedly strengthening the physical constraint force (crystalline constraint effect) on the amorphous chains existing between crystals. Consequently, the intrinsic deformability (ductility) of the matrix was suppressed and the strain at break decreased drastically from approximately 0.040 to approximately 0.025: it can be inferred that dramatic enhancement in macroscopic stiffness (elastic modulus) and yield stress occurred.

### 4.2. Microscopic Physics of Strength Reduction and Repair Time Dependence in Repetitive “Damage–Thermal Repair” Cycles

The most important physical phenomenon to be examined for this study is the fact that in the first repair, both conditions (120 s/1800 s) completely recover strength to a level equivalent to the un-treated material (103–105 MPa). However, as the cycles are repeated, the strength decreases step-by-step, resulting in a dramatic bifurcation in mechanical properties after the third repair between the 120 s condition (approximately 30 MPa) and the 1800 s condition (approximately 59 MPa). This macroscopic strength trend is discussed from three perspectives: reptation dynamics in the partially melted state of the polymer, annealing-time-dependent higher-order structure reorganization, and the separation process between “thermally repairable matrix phase damage” and “extremely difficult to thermally repair fiber/resin interface damage”.

#### 4.2.1. Delay of Molecular Chain Interdiffusion Based on Reptation Theory and Its Time Dependence

For this study, the interface repair of thermoplastic resin is discussed based on the Reptation Theory proposed by De Gennes [13]. A polymer main chain heated near the glass transition temperature or melting point (melting peak top: approximately 170 °C) initiates Brownian motion (reptation) along a spatial constraint tube formed by surrounding polymer chains. The time necessary for a polymer chain to escape from its initial tube completely, cross the opposing damaged interface, and to reconstruct the intrinsic entanglement density of the material is designated as the reptation time τ_r_. When the molecular weight is denoted as M, it is known to exhibit strong dependence that is proportional to the cube of M (M^3^), as shown in Equation (5).

τ_r_∝M^3^ (5)

For quantitative evaluation of this relaxation time scale for the commercial polypropylene (PP) matrix used for this study (Daicel Polymer Ltd., PG6N1), a representative weight-average molecular weight of M_w_ ≒ 3.0 × 10^5^ g/mol was adopted from data referred from the relevant literature [18]. In a fully isotropic melt state (e.g., at 200 °C), the fundamental reptation time τ_r_ for PP of this molecular weight class is estimated as being on the order of 10^−1^ to 10^0^ s based on viscoelastic melt relaxation parameters and zero-shear viscosity scaling.

However, as demonstrated by the DSC thermogram presented in Figure 3, the thermal repair temperature adopted for this study (160 °C) corresponds to the onset region of crystal melting (a sub-melting, partially molten state). In this temperature regime, rigid un-melted crystalline lamellae remain intact and act as physical crosslinks and impenetrable spatial barriers (crystalline constraint effect), severely restricting the lateral and reptational mobility of amorphous chains. Consequently, the apparent self-diffusion coefficient is suppressed dramatically, extending the effective reptation time (τ_r,eff_) under crystalline constraints by several orders of magnitude (τ_r,eff_ ≫ 10^2^ s) relative to the fully unconstrained melt state.

Under the short heating condition of 120 s, the holding duration t is markedly insufficient relative to this prolonged effective reptation time (t ≪ τ_r,eff_). Consequently, the process by which amorphous PP chains deeply interdiffuse across the damaged interface to regenerate bulk entanglement density, as well as the viscoelastic flow required to re-wet the debonded glass fiber surfaces physically, is forcibly terminated at an incomplete initial stage. Although local matrix crazes (reversible damage) are closed because of short-range segmental relaxation, unhealed interfacial debonding (irreversible defects) persists. By contrast, extending the annealing time to 1800 s provides an adequate thermal flow window (t ~ τ_r,eff_), enabling molecular chains to overcome the kinetic barriers imposed by crystalline constraints, achieve deeper interfacial re-entanglement, and promote physical re-adhesion onto the fiber surface.

#### 4.2.2. Overcoming the Trade-Off Between Crystal Growth and Reptation Under the 1800 S Condition

By contrast, when applying the prolonged heating condition of 1800 s, the crystallinity index increased continuously to 0.42 (1st), 0.44 (2nd), and further to 0.49 (3rd) along with repetition of the annealing process, indicating that considerable growth and reorganization of the lamellar structure progressed. Generally, when the degree of crystallinity in higher-order structures increases continuously in this manner, the nanoscale crystalline constraint effect becomes dominant. Therefore, the free volume of amorphous chains decreases. Moreover, the reptation motion (diffusion liquidity) is delayed and inhibited even more severely (a trade-off between stiffness improvement and polymer mobility).

However, by securing an extremely sufficient heating/holding time of 1800 s, this thermodynamic barrier of motion delay caused by crystalline constraint was overcome. This barrier made it possible to allow molecular chain interdiffusion across the interface to proceed sufficiently deep, and to allow the high-viscosity PP matrix to flow into the debonded gaps around the fibers. As demonstrated by fracture mechanics studies by Jud, Kausch, and Williams of polymer crack repair and welding, sufficient heat treatment time is an indispensable requirement for completely recovering physical entanglement binding force and reorganizing strong interfacial bonding [22]. For this reason, in the long-time annealing group (1800 s), the synergistic effects of retaining the intrinsic stiffness and elastic modulus of the bulk matrix attributable to increased crystallinity, together with deep re-entanglement formation across the interface and physical re-adhesion to fibers, operated. Consequently, even after the third repair, high practical strength of approximately 59 MPa was maintained successfully.

### 4.3. Separation Model of Irreversible Damage (Debonding) at the Fiber–Resin Interface and Matrix Structural Failure

**Figure 10:**
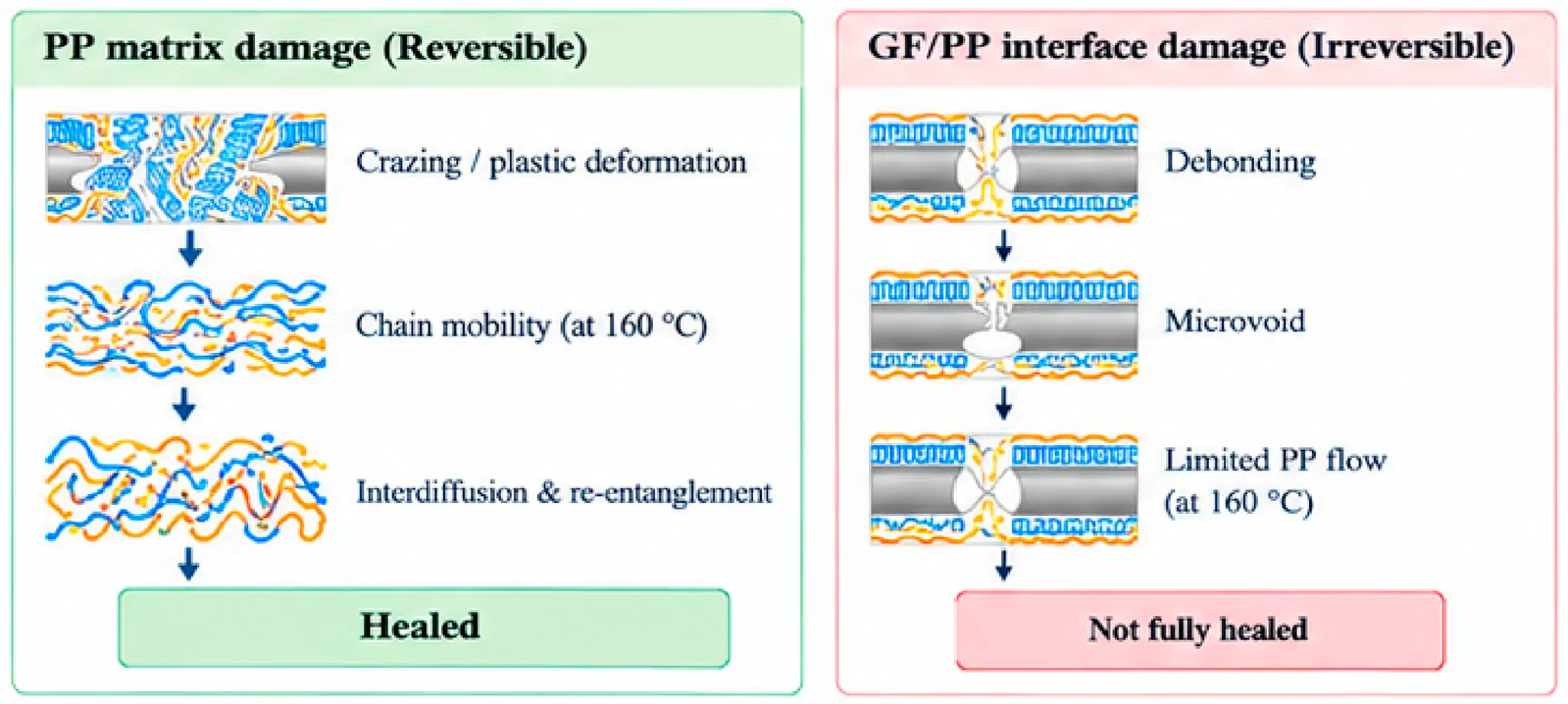
Schematic illustration of the hypothesized repair mechanisms during thermal annealing (at 160 °C) for different damage modes in glass fiber-reinforced polypropylene (PP/GF) composites.

The mechanism discussed in Section 4.2 is regarded as sufficient to explain matrix damage recovery across varying annealing times, but the behavior of flexural strength demonstrates that even if the matrix recovers sufficiently, full strength is not restored. Therefore, for this study, we propose a qualitative schematic model of microstructural damage behavior based on changes in macroscopic mechanical properties (a gradual decrease in flexural strength and fracture strain) associated with repeated “damage–thermal repair” cycles, as well as structural analysis results (FT-IR and X-ray CT). Based on the macroscopic mechanical property trends (step-wise degradation in flexural strength and strain at break) and non-destructive structural analyses (FT-IR and micro-CT), a hypothesized schematic model of the microstructural damage and repair mechanism is proposed as presented in Figure 10. Sub-micron interfacial debonding and microvoid growth occur below the spatial resolution limit of the micro-CT setup. Therefore, the mechanisms depicted in Figure 10 represent a conceptual model proposed to rationalize the observed macroscopic mechanical degradation based on molecular reptation theory and spectroscopic trends, rather than direct imaging. Although direct spatial resolution of sub-micrometer interfacial debonding (microvoids) exceeds the contrast threshold of the present micro-CT setup, this model physically rationalizes the observed recovery limits. In this framework, matrix crazes and plastic deformation are treated as thermally reversible defects, whereas fiber–matrix debonding is represented as a thermally persistent, irreversible structural defect.

#### 4.3.1. Reversible Reparability of Crazes and Plastic Deformation in the Matrix Phase

The damage introduced into the PP matrix phase by three-point bending load is mainly local plastic deformation and “crazes” accompanied by voids and fibrils as crack precursors. These physical distortions and fine voids in polymer chains within the amorphous region are fundamentally reversible defects that can be thermally self-repaired (healed).

When a partial melting temperature of 160 °C is applied, molecular mobility in the amorphous region is activated rapidly while preserving the crystalline skeleton. According to De Gennes’ Reptation Theory [13], amorphous chains initiate Brownian motion (self-diffusion) and easily form interdiffusion and re-entanglement across the damaged interface. As a result, the original continuity and soundness of the matrix are restored quickly. After the first thermal repair, mechanical strength recovered to a level equivalent to the initial strength of the untreated material (103–105 MPa) under both 120 s and 1800 s conditions. This recovery is possible, probably because crazes and fine plastic deformation generated in the matrix phase were self-repaired (thermally closed) efficiently by thermal press annealing, although interfacial damage (described later) remained in trace amounts.

#### 4.3.2. Thermal Non-Reparability of Debonding at the Fiber/Resin Interface

However, the primary governing factor affecting the macroscopic mechanical strength of the entire composite material is the “load transfer efficiency (interfacial shear strength)” at the fiber–matrix interface. When flexural deformation greatly exceeds the elastic limit, debonding between inorganic glass fibers and organic PP resin is assumed to occur extensively near the skin layer, where stress is concentrated.

This fiber–resin interface debonding is “irreversible damage” where physical–chemical bonds at the interface (such as chemical bonding by silane coupling agents) are broken by mechanical external force. Unlike crazes inside the matrix polymer, it inherently possesses the property of being unrecoverable merely by thermal motion and physical relaxation of polymer chains.

Because glass fiber itself is an inert substance showing no melting or geometrical change even in a 160 °C heating environment, rejoining this debonded surface requires the partially melted, high-viscosity PP matrix to achieve physical “re-adhesion/rewetting” against micro-voids around the fibers via thermal expansion and local viscoelastic flow. However, PP in the partially melted state at 160 °C still exhibits extremely high melt viscosity. Especially in short-time (120 s) heat treatment, the flow time to wet the fiber interface sufficiently is wholly lacking. As a result, although crazes in the matrix itself are closed and repaired, most debonded regions at the fiber–resin interface remain irreversibly inside the material as “unrepaired micro-voids (interfacial defects)” without achieving re-adhesion.

#### 4.3.3. Impossibility of Complete Recovery and Scenario of Step-by-Step Strength Collapse

The contrast model above, incorporating “matrix reversible repair” and “interface irreversible fracture,” clearly organizes the essence of the mechanical behavior in this study.

First, we address “the reason why it does not recover to the intrinsic maximum strength (122 MPa) of the initially annealed material completely, even after thermal repair”: The initially annealed material (annealed) acquired an extremely high strength potential of 122 MPa because of matrix stiffness and because of the increased yield stress with crystallization during annealing. However, in specimens subjected to damage once (1st repair), even if the matrix reaches a similar degree of crystallinity (0.37) and increases bulk strength, a portion of the irreversible debonding (micro-defects) generated at the fiber interface remains as unrepaired defects. Under external loading, stress concentrates locally around these unrepaired points (voids). Therefore, smooth load transfer from matrix to fiber, which is the intrinsic reinforcement mechanism of the material, is hindered, causing macroscopic fracture to initiate early at a lower level of stress (103–105 MPa) before demonstrating the original potential of the annealed material (122 MPa).

Second, “the reason why strength decreases step-by-step with repeated cycles and shows a dramatic property bifurcation at the third cycle”: When second and third bending damage (large flexural deflection) is applied to the repaired specimen, new mechanical stress preferentially and non-uniformly concentrates around the “fiber/resin interface debonding regions (micro-voids)” that remained unrepaired inside the material from the preceding cycle. Because of this stress concentration, interfacial debonding re-propagates easily and expands with much less energy than in the preceding cycle, growing easily into macroscopic delamination linked directly to material fracture by coalescence with adjacent unrepaired voids.

Under the short-time (120 s) condition, because this interface rewetting/adhesion process ends near zero, the accumulation rate of “unrepaired debonding voids” increases exponentially with each cycle. After the third repair, the fiber reinforcement–load-transfer effect collapses completely. Mechanical properties are thought to be lost entirely down to a level equivalent to the mechanical strength of unreinforced PP matrix alone (approximately 28–30 MPa).

In contrast, when applying the long-time (1800 s) condition, an extremely sufficient thermal flow time of 30 min (1800 s) was secured, markedly increasing the opportunity for polymers to “re-adhere” physically to the fiber interface. As a result, chemical and physical re-adhesion of interfacial debonding was promoted to deeper regions, suppressing the “accumulation rate of unrepaired voids” within cycles to the absolute minimum. Consequently, even after three repeated load cycles, high strength of approximately 59 MPa could be maintained.

### 4.4. Geometrical and Orientational Stability of Sub-Melting Temperature Thermal Repair

As a unique characteristic of this study, even when repetitive “damage–thermal repair” cycles were applied, the following three points were demonstrated.

1. The average fiber orientation angle in the skin layer region was maintained within a constant range of 27° to 33°, with an orientation distribution range of 5° to 55°.
2. Hermans orientation index S remained within the extremely stable range of −0.19 to −0.26, exhibiting no loss of molecular orientation anisotropy.
3. Poisson’s ratio remained invariant as 0.38–0.44, maintaining the elastic deformation mechanism.

These measurement results clearly support the validity of the 160 °C thermal repair process window selected in this study from the viewpoint of polymer structure theory. Because 160 °C is a partial melting region below the PP crystal melting peak (170 °C), the strong crystalline skeleton (the majority of the lamellar structure) supporting the bulk shape remains un-melted, thereby preserving the structural frame of the material.

Therefore, macroscopic melt flow of the matrix resin (i.e., deconsolidation accompanied by sufficient void expansion and dimensional changes) is suppressed effectively. The process demonstrated an extremely practical benefit: structural defects can be repaired by selectively driving only local self-diffusion (reptation) in the amorphous region, while completely preserving the geometrical anisotropy of glass fibers and the orientational order of polymer chains imparted during molding.

Regarding the effective Poisson’s ratio (υ_eff_), the as-molded specimen exhibited a value of 0.38, whereas the initial annealed specimen was 0.42. Across all subsequent repetitive damage–thermal repair cycles, υ_eff_ remained within the remarkably stable range of 0.38–0.44.

From a mechanical physics standpoint, these values (0.38–0.44) are slightly higher than the classical linear-elastic Poisson’s ratio of isotropic solid polypropylene. This difference is attributed to two competing micro-mechanical phenomena occurring in the skin layer near flexural yielding.

1. As the matrix approaches its flexural yield stress (σ_fy_), localized micro-plastic flow is activated, causing the incremental Poisson’s ratio of the polymer phase to shift toward the incompressible limit (υ → 0.5).
2. Simultaneously, the initiation of micro-crazes and interfacial debonding induces local volumetric expansion, whereas the rigidly aligned glass fibers in the skin layer exert mechanical lateral constraints.

The balance between matrix plastic softening (which increases υ_eff_) and micro-structural void evolution or fiber constraint governs the apparent value within 0.38–0.44. Most importantly, the high stability of υ_eff_ across repetitive repair cycles proves that sub-melting thermal annealing restores the stress transfer capability and bulk lateral deformation characteristics of the skin layer without inducing severe structural degradation or macro-void coalescence that would otherwise alter the lateral elasticity of the composite drastically.

### 4.5. Engineering Guidelines for Practical Material and Process Design

The academic findings obtained in this study provide the following specific process engineering guidelines toward sustainable, long-life, and maintenance-free structural design of thermoplastic composite materials (GFRTP).

- To perform repair in a short time (120 s) on-site, conducting precise temperature control even closer to the melting point than 160 °C (e.g., 163–165 °C) is necessary to release crystalline constraints moderately and shorten the reptation time τ_r_, or to apply external compressive pressure (thermal re-molding) simultaneously to assist the wetting and adhesion of polymer chains physically.
- To eliminate thermal degradation or deconsolidation of the entire structure and to complete interfacial reptation motion (interdiffusion) quickly, development into smart maintenance techniques is expected. This development includes applying localized and selective heating, such as “Joule resistance heating” using conductive fibers or “induction heating (IH)” for instantaneous melting only of the damaged interface above the melting point.
- Beyond process control (temperature, time, pressure), it is also important from the viewpoint of matrix resin material composition to introduce mechanisms that “relieve strain remaining in and around crystalline regions”. For example, future research topics include investigating changes in higher-order structure and strain relaxation behavior when blending elastomer components (TPO, EPR, etc.) into the PP matrix, as well as the consequent effects on thermal repair capability.

## 5. Conclusions

This study systematically evaluated PP/GF composites, which are glass fiber reinforced thermoplastics (GFRTP), to elucidate the effects of repetitive “damage–thermal repair” cycles on their mechanical properties and hierarchical structures. This study simulated long-term operation and maintenance of actual structural components. Evaluation was based on a model separating the recovery process into the “thermal recovery of reversible damage in the matrix” and the “non-recovery of irreversible damage at the fiber–resin interface.” The main findings of this study are summarized as follows:

1. Complete Initial Recovery: Sub-melting thermal annealing at 160 °C restored the flexural strength of damaged PP/GF composites to 103–105 MPa after the first cycle (100% recovery relative to the as-molded state).
2. Dwell Time Dependence: Extended repair time (1800 s) significantly outperformed short treatment (120 s) during repetitive cycles, retaining 59 MPa (57.3% of initial strength) after three cycles by allowing sufficient time for matrix re-wetting onto fibers under crystalline constraints.
3. Structural Anisotropy Retention: Micro-CT and FTIR analyses confirmed that macroscopic fiber orientation and molecular chain alignment were statistically preserved across cycles (*p* > 0.05).
4. Mechanistic Framework: A conceptual repair framework was established, demonstrating that while matrix crazing is thermally reversible, irreversible interfacial debonding governs the multi-cycle recovery limit.

These findings highlight that complementary process control of temperature and repair duration (or pressure) is crucially important for the sustainable operation (extending service life and implementing a circular economy) of thermoplastic composites. Looking forward, expansion of this approach to advanced local thermal stimulation techniques such as non-contact induction heating (IH) or local resistance heating via conductive nanonetworks that are integrated intelligently with structural health monitoring (SHM) technology can be expected to provide clear guidelines for establishing smart, low-energy structural regeneration technologies.

## Data Availability

The data presented for this study are sufficient for the purposes of this investigation. They are available for further analysis upon request from the corresponding author.
